# A Fast Firefly Algorithm for Function Optimization: Application to the Control of BLDC Motor

**DOI:** 10.3390/s21165267

**Published:** 2021-08-04

**Authors:** Smail Bazi, Redha Benzid, Yakoub Bazi, Mohamd Mahmoud Al Rahhal

**Affiliations:** 1 Department of Science and Technology, University Center of Abdelhafid Bousouf Mila, Mila 43000, Algeria; s.bazi@centre-univ-mila.dz; 2 Research Laboratory of Electromagnetic Induction and Propulsion Systems, Department of Electrical Engineering, University of Batna 2, Batna 05000, Algeria; 3 Laboratoire d’Automatique Avancée et d’Analyse des Systèmes: LAAAS, Department of Electronics, University of Batna 2, Batna 05000, Algeria; r.benzid@univ-batna2.dz; 4 Computer Engineering Department, College of Computer and Information Sciences, King Saud University, Riyadh 11543, Saudi Arabia; 5 Applied Computer Science Department, College of Applied Computer Science, King Saud University, Riyadh 11543, Saudi Arabia; mmalrahhal@ksu.edu.sa

**Keywords:** Fast Firefly Algorithm, optimization, benchmark functions, BLDC motor, PI controller, nature inspired algorithm

## Abstract

Firefly Algorithm (FA) is a recent swarm intelligence first introduced by X.S. Yang in 2008. It has been widely used to solve several optimization problems. Since then, many research works were elaborated presenting modified versions intending to improve performances of the standard one. Consequently, this article aims to present an accelerated variant compared to the original Algorithm. Through the resolving of some benchmark functions to reach optimal solution, obtained results demonstrate the superiority of the suggested alternative, so-called Fast Firefly Algorithm (FFA), when faced with those of the standard FA in term of convergence fastness to the global solution according to an almost similar precision. Additionally, a successful application for the control of a brushless direct current electric motor (BLDC) motor by optimization of the Proportional Integral (PI) regulator parameters is given. These parameters are optimized by the FFA, FA, GA, PSO and ABC algorithms using the IAE, ISE, ITAE and ISTE performance criteria.

## 1. Introduction

Optimization is one of the methods that seek to solve complex problems in engineering or other fields. The objective of optimization is to locate the optimal value of a cost function in a well-defined research space under different constraints [1]. Among the techniques used, in optimization, are those of swarm intelligence algorithms which are nature-inspired algorithms, these optimization techniques have spread over the past two decades [2]. Thus, the significant performance of swarm intelligence algorithms compared to other conventional optimization methods motivates researchers and are still to be attractive to exploit them in several complex optimization problems at different fields [3]. These algorithms operate on two different search properties: exploitation and exploration, where exploration scans the entire search space and prevents the algorithm from falling into the local optima, while exploitation ensures the efficiency of the search and the convergence of the algorithm towards the optimal solution [4]. Since the appearance of Genetic Algorithm [5], many optimization algorithms have been proposed such as Ant Colony Optimization [6,7], Artificial Bee Colony (ABC) [8,9], Particle Swarm Optimization (PSO) [10,11] Modified Particle Swarm Optimization [12] Cuckoo Search (CS) [13,14], Bat Algorithm (BA) [15,16], Gray Wolf Optimizer (GWO) [17,18], Firefly Algorithm [19,20] and so on.

Recently, Firefly Algorithm is one of the famous swarm intelligence algorithms for optimization problems that have been introduced in 2008. Due to its ease of design, implementation and flexibility in nature, it has become popular in the field of optimization and has been widely applied to diverse engineering optimization problems such as in [21,22]. Despite all these advantages, it has drawbacks such as the problem of local minima and it was unable to guarantee a balance between exploration and exploitation [2,23]. Therefore, several improvement algorithms have been proposed to overcome such drawbacks which make them more widely applied successfully in engineering like optimizing Proportional Integral Derivative (PID) parameters in machine control [24,25,26,27,28].

The PID controller and its variants are mainly used in control process to have a better dynamic performance of the controlled systems. Therefore, the optimal value of the corrector parameters is needed. In this context, the choice of controller gains has become an optimization problem [29]. FA and rival algorithms were successfully applied in the optimization of the parameters of PID mainly in electrical engineering and other fields [30]. One of the prominent applications in electrical engineering is the control of BLDC motor driven by a tuned and optimized PID. However, a BLDC motor is developed on the basis of Brushed DC motor and it is one of the special electrical synchronous motor. It is driven by DC voltage, but current commutation is obtained by solid-state switches. The commutation time is fixed by the rotor position which is detected by hall sensor position [31].

It is noticeable that BLDC motor has the advantages that are: high efficiency, long operating time, low noise, small size and well speed–torque features. In general, it has a great improvement in automotive, aerospace and industry of engineering and so on. Therefore, its use has been exposed to many types of load disturbances. Conventional control methods cannot resist these alterations and lose their precision. Thus, it was necessary to implement advanced control techniques to solve this problem, especially those based on the artificial intelligence, such as: fuzzy control [32,33], neural control [34,35], Genetic Algorithm (GA) control [36,37], PSO control [38], BAT control [31] and recently, FA control and Improved Firefly Algorithm (IFA) or Modified Firefly Algorithm (MFA) [24,25,26,27,28]. These methods are based essentially on the optimization of the PID corrector parameters and its derivatives to obtain optimal performance.

In this paper, we propose an improved version of the FA for function optimization by reducing the search space. We apply this method to several benchmark problems and also to the design of a controller for BLDC motor. The paper contains two experimental parts, the first concerns the search for the global optimum of several benchmark functions according to the FA and FFA algorithms and then a comparative study is carried out. In order to consolidate its efficiency, a second application of PI parameters’ optimization for the BLDC motor control is achieved through simulation in the MATLAB platform. This application used the FFA, FA, GA, PSO and ABC algorithms according to the IAE, ISE, ITAE and ISTE performance criteria, to test the competitiveness of the FFA algorithm. Finally, by comparison of the obtained results, it is found that the performances of the FFA are better than those of the other algorithms and it can be concluded that this new algorithm can be a valid concurrent meta-heuristic optimization method.

The paper is organized as follows. Section 2 introduces the mathematical background of the standard FA and the suggested FFA. In Section 3, the two algorithms are compared through optimum finding of several standard test functions. The mathematical model of BLDC motor and the PI controller with description of the experimental results are presented in Section 4. Finally, drawn conclusion summarizing the achieved work is given in Section 5.

## 2. Firefly Algorithm and Proposed Fast Firefly Algorithm

### 2.1. Standard Firefly Algorithm 

Firefly Algorithm is inspired by the natural behavior of fireflies by using their self-luminosity to get closer to each other in the dark. Three assumptions have been suggested by Yang to clarify the behavior of fireflies [19,20]. Firstly, all fireflies are unisex. Thus, each firefly can be attracted to other fireflies regardless of gender. Secondly, the attractiveness is linked to the intensity which is a function of the distance between the firefly concerned and the other fireflies. The attractiveness decreases as the distance increases. Finally, the luminosity or the luminous intensity of a firefly is given by the value of the cost function of the problem posed. Mathematically, the FA algorithm can be given by the following equations [19].

The light intensity of a firefly is given by Equation (1).

(1) $$I(r)=I_{0}\exp(-\gamma .r_{ij})$$

where: *γ* is the absorption coefficient and (*I*_0_) is the initial value at (*r* = 0).

The attractiveness is expressed by Equation (2) where *β*_0_ is the initial value at (*r* = 0):

(2) $$\beta =\beta_{0}\exp(-\gamma .r_{ij}^{m})\;,\;m\geq 1$$

Equation (3) evaluates the distance between two fireflies *i* and *j*, at positions *x_i_* and *x*_j_, respectively, and can be defined as Cartesian distance. Where *x_ik_* is the *k*th element of the spatial coordinate *x_j_* of the *i*th firefly and D denotes the dimensionality of the problem [19].

(3) $$r_{ij}=\left|r_{i}-r_{j}\right|=\sqrt{\sum_{k=1}^{D}{\left(x_{ik}-x_{jk}\right)}^{2}}$$

The motion equation of the *i^th^* firefly to *the j^th^* one is determined by Equation (4).

(4) $$x_{i}(t+1)=x_{i}(t)+\beta (x_{j}(t)-x_{i}(t))+\alpha (rand-0.5)$$

where *x_i_*(*t* + 1) is the position of firefly *i* at iteration *t* +1 displacement. As it can be seen, the first part of the right side of Equation (4) is the position of firefly *i* at iteration *t*, the second term is relative to the attractiveness and the last one is randomization (blind flying if there is no light) where α is the random walk parameter *α* ∈ [0,1), [19]. 

The FA Algorithm 1 is given as follows [19]:
**Algorithm 1. Firefly Algorithm***Initialization of the parameters of FA (Population size, α, β_o_, γ and the number of iterations).* *The Light intensity is defined by the cost function f(x_i_) where x_i_(i = 1,…,n).* *While (iter < Max Generation).* *         for i = 1:n (all n fireflies)* *           for j = 1:n (all n fireflies)* *             if (f(x_i_) < f(x_j_)), move firefly i towards j,* *             end if.* *             Update attractiveness β with distance r.* *             Evaluate new solution and update f(x_i_) in the same way as (4).* *           end for j* *         end for i* *rank the solutions and find the best global optimal.* *end while.* *Show the results.*

### 2.2. Fast Firefly Algorithm

It is worth noting that the original algorithm of Xin-She Yang performs (Max generation *n.n*) tests. However, in the proposed version, (*K*.*n*) tests only are performed, where *K* is an integer. It means that the conventional one is hugely time consuming when compared to the suggested one. The proposed Algorithm 2 is summarized as follows:
**Algorithm 2. Fast Firefly Algorithm***While (iter < Max Generation)* *         for k = 1:K.n (all n fireflies) // Here it is the first modification* *                 i = rand(n) // Here it is the second modification* *                 j = rand(n) // Here it is the third modification* *                 if (f(xi) < f(xj)), move firefly i towards j,* *                 end if.* *                 Update attractiveness β with distance r.* *                 Evaluate new solution and update f(xi) in the same way as Equation (4).* *                 Modify the new position obtained by Equation (4) according to Equation (5).* *                 end for k* *rank the solutions and find the best global optimal.* *end while.* *Show the results.*

As above mentioned, the new position obtained by Equation (4) is modified according to Equation (5):

(5) $$x_{i}(t+1)=\alpha .x_{i}(t)$$

It should be noted that the values of *α* and *γ* are given empirically in the original version according to each test function, *β*_0_ is equal to unity. However, on the other hand, the *α* in FFA is taken equal to:

(6) α=exp(−10.iter/(iter+100))

where the convergence is reached easily and *γ* still chosen equal to 1. The randomization parameter *α* is reduced exponentially from a maximum value to a minimum value according to successive iterations instead of keeping it constant; with this injected artifice, we can maintain the research balance between the exploitation and the exploration of the proposed algorithm and it can give better results than its rival FA [4].

In the original version of the FA, the technique of updating the motion of fireflies can be improved to be more faster. Thus, it is beneficial for each firefly in the swarm to find a promising region by reorienting its motion in order to easily reach the overall optimum. Consequently, the updated term is redirected to have a better exploration and exploitation of the algorithm and the speed of its convergence is, thus, guaranteed [1,39].

The essence of the proposed method is the reduction of the search space (exploration) while keeping the search efficiency satisfactory to reach the optimal solution. It means that (*K.n*) evaluated tests were found clearly sufficient to obtain the optimal solution for the large number of benchmark functions and other applications [40]. 

## 3. Simulation Results and Analysis

### 3.1. Benchmark Functions 

Standards’ functions are essential to prove and compare the characteristics of optimization algorithms. The most terms of evaluation are: The convergence speed and the precision. Hence, 12 different test functions are used to compare the performance of the original algorithm FA and the proposed one FFA according to the previously mentioned evaluation terms. The used test functions are listed in Table 1, highlighting the variables, ranges and values of the global optimum to reach [41,42].

### 3.2. Parameter Settings

The parameter settings of FA and FFA are showed in Table 2.

### 3.3. Functions’ Experimental Results 

The two algorithms are applied to minimize a set of test functions of dimensions 2D, 10D, 20D and 30D, respectively. The experimental environment is the MATLAB R2017a software, the CPU is an E5700@3.00 GHZ, the RAM is of size 6 GB. To compare their performance, minimum, mean, standard deviation and the computational time are taken over 10 runs. For each function, the two algorithms operate independently. The results of the optimization are summarized in Table 3.

In terms of precision of convergence towards the global optimum, by visualizing the results in Table 3, it can be seen that the mean and the standard deviation of the reached optimum, after 10 runs for each test function, of FFA in all dimensions are better than of FA. 

Concerning the convergence fastness to the global optimum, it can be clearly remarked, from extensive simulation tests, that the proposed method outperforms the original one and it is significantly faster (see Table 3). Accordingly, the average speed up ratio, when applying the two algorithms on the 12 test functions, is 12:1, which confirms the effectiveness of the suggested technique.

It is worthy to note that the speed up ratio is defined by:

(7) $$SR=\frac{t_{FA}}{t_{FFA}}$$

where *t_FA_* is the execution time of the original algorithm FA, and the *t_FFA_* is the execution time of the proposed one FFA.

**Figure 1:**
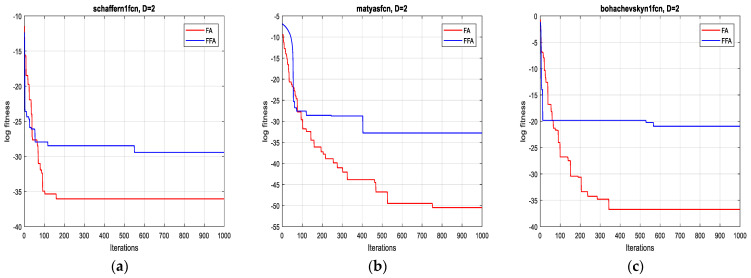
Convergence curves of FFA and FA for the functions: (**a**) F1; (**b**) F2 and (**c**) F3 (2D).

**Figure 2:**
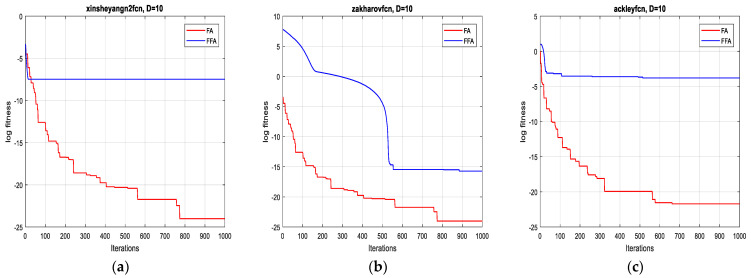
Convergence curves of FFA and FA for the functions: (**a**) F4; (**b**) F5 and (**c**) F6 (10D).

**Figure 3:**
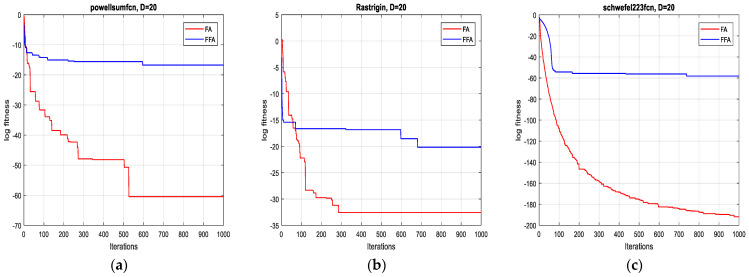
Convergence curves of FFA and FA for the functions: (**a**) F7; (**b**) F8 and (**c**) F9 (20D).

**Figure 4:**
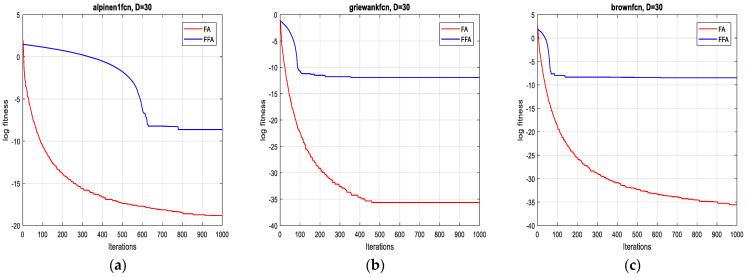
Convergence curves of FFA and FA for the functions: (**a**) F10; (**b**) F11 and (**c**) F12 (30D).

As is shown in Figure 1, Figure 2, Figure 3 and Figure 4 below, the proposed algorithm reaches all solutions of all test functions with high precision outperforming, accordingly, those obtained from the standard one.

**Figure 5:**
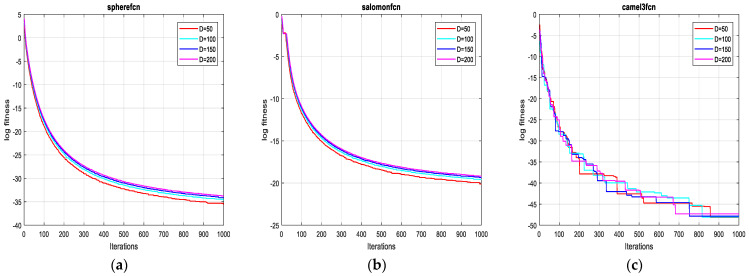
Convergence curves of FFA for the functions: (**a**) F13; (**b**) F14 and (**c**) F15 on 50D, 100D, 150D and 200D.

As can be seen from Table 3, the proposed algorithm is more unbiased (the statistical expected value of obtained cost function of FFA is more tending to the theoretical value than FA) and more consistent (the standard deviation of obtained cost function when applying FFA is more tending to 0 than the FA). The reported remarks hold for the twelve test functions as previously shown in Table 3 for dimensions 2D, 10D, 20D and 30D, respectively. For more convincing, robustness and stability of FFA in higher dimensions are evaluated by using the test functions (F13, F14 and F15) for dimensions 50D, 100D, 150D and 200D, respectively. Table 4 gives the results of these tests with a 10 times run for each test function. Finally, it can be concluded that the stability of FFA is not affected by increasing significantly dimensions (high precision remains obtained). The graphs of Figure 5 reflect these results.

## 4. Application for the Control of Brushless DC Motor

### 4.1. Description

BLDC motor is a permanent magnet synchronous motor that has trapezoidal Back- EMF and an almost rectangular current. It uses position detectors and an inverter to control the armature currents. It becomes popular for industrial applications because of its high efficiency, reliability, noiseless operation, low maintenance and an optimized volume. BLDC motors are available in several different configurations, but three-phase is the most common type due to its high speed and low torque ripple [43].

**Figure 6:**
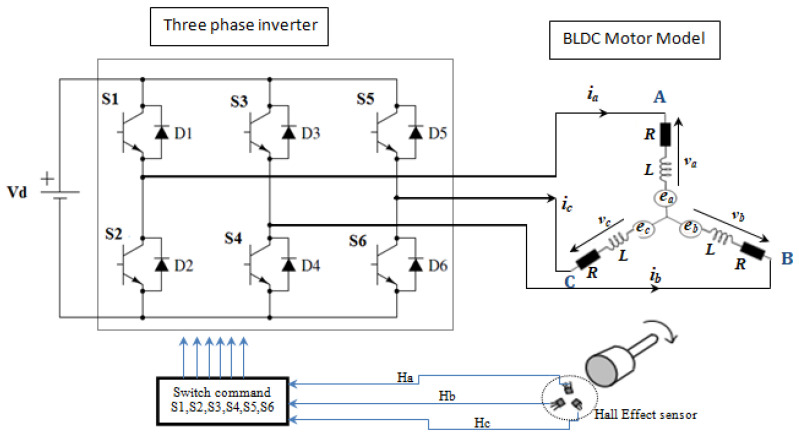
Drive model of a BLDC motor [43,44,45,46,47].

The drive model of a BLDC motor is shown in Figure 6. It is divided into two blocks. The first one is the inverter and the second is the BLDC motor. Accordingly, the BLDC motor is powered by a six-switch inverter where, for each control step, two phases operate simultaneously while the third is eliminated. Note that the signals of the Hall Effect position sensor (Ha, Hb, Hc) shifted by 120°, electrically govern these switches by generation of the pulses (S1,…,S2) at every 60° electrical angle [43,44,45].

### 4.2. Mathematical Modeling of a BLDC Motor

By consideration of the symmetry of the phases, it is assumed that the three phases’ resistances are identical as well as the inductances. Consequently, the equations describing the model of the equivalent circuit of the motor are [43,44,45]:

(8) $$v_{a}=Ri_{a}+L\frac{d}{dt}i_{a}+e_{a}$$

(9) $$v_{b}=Ri_{b}+L\frac{d}{dt}i_{b}+e_{b}$$

(10) $$v_{c}=Ri_{c}+L\frac{d}{dt}i_{c}+e_{c}$$

Then, the line voltage equation can be obtained by subtraction of the phase voltage equation as:

(11) $$v_{ab}=R\left(i_{a}-i_{b}\right)+L\frac{d}{dt}\left(i_{a}-i_{b}\right)+e_{a}-e_{b}$$

(12) $$v_{bc}=R\left(i_{b}-i_{c}\right)+L\frac{d}{dt}\left(i_{b}-i_{c}\right)+e_{b}-e_{c}$$

(13) $$v_{ca}=R\left(i_{c}-i_{a}\right)+L\frac{d}{dt}\left(i_{c}-i_{a}\right)+e_{c}-e_{a}$$

where:

*R*: resistance of a stator phase [Ω].
*L*: inductance of a stator phase [H].
*v_a_, v_b_* and *v_c_* are the stator phase voltages [V].
*v_ab_, v_bc_* and *v_ca_* are the stator phase to phase voltages [V].
*i_a_, i_b_* and *i_c_* are stator phase currents [A]
*e_a_, e_b_ and e_c_* are motor Back-EMFs [V].

The relationship between phase currents is given by the equation:

(14) $$i_{a}+i_{b}+i_{c}=0$$

Since each voltage is a linear combination of the other two voltages, two equations are sufficient. Using relation 14, Equations (11) and (12) become [44]:

(15) $$v_{ab}=R\left(i_{a}-i_{b}\right)+L\frac{d}{dt}\left(i_{a}-i_{b}\right)+e_{a}-e_{b}$$

(16) $$v_{bc}=R\left(i_{a}+2i_{b}\right)+L\frac{d}{dt}\left(i_{a}+2i_{b}\right)+e_{b}-e_{c}$$

The equation of mechanical part represents as follows:

(17) $$T_{e}=k_{f}\omega_{m}+J\frac{d\omega_{m}}{dt}+T_{L}$$

(18) $$\omega_{m}=\frac{d\theta_{m}}{dt}$$

where:

*T_e_* and *T_L_* are the electromagnetic torque and the load torque [Nm].
*J* is the rotor inertia, *k_f_* is a friction constant and ωm is the rotor speed [rad/s].

The Back-EMF and electromagnetic torque can be expressed as:

(19) $$e_{a}=k_{e}\omega_{m}F(\theta_{e})$$

(20) $$e_{b}=k_{e}\omega_{m}F\left(\left(\theta_{e}-\frac{2\pi }{3}\right)\right)$$

(21) $$e_{c}=k_{e}\omega_{m}F\left(\left(\theta_{e}-\frac{4\pi }{3}\right)\right)$$

where:

*k_e_* is the Back-EMF’s constant.
$\theta_{e}$ is equal to the rotor angle ($\theta_{e}$= *p*.$\;\theta_{m}$/2), $\theta_{m}$ the mechanic angle and *p* the number of pole pairs. *F*($\theta_{e}$) is trapezoidal waveform of Back-EMFs.

Thus, the torque equation can be defined as:

(22) $$T_{e}=\frac{\left(e_{a}i_{a}+e_{b}i_{b}+e_{c}i_{c}\right)}{\omega_{m}}=\frac{k_{t}}{2}\left[F\left(\theta_{e}\right)i_{a}+F\left(\theta_{e}-\frac{2\pi }{3}\right)i_{b}+F\left(\theta_{e}-\frac{4\pi }{3}\right)i_{c}\right]$$

*kt*: the torque constant.

Therefore, the function *F*($\theta_{e}$) is a function of rotor position, which gives the trapezoidal waveform of Back-EMF. One period of function can be written as:

(23) $$F(\theta_{e})=\left\{\begin{array}{cc} 1 & 0\leq \theta_{e}<\frac{2\pi }{3}\\ 1-\frac{6}{\pi }(\theta_{e}-\frac{2\pi }{3}) & \frac{2\pi }{3}\leq \theta_{e}<\pi \\ -1 & \pi \leq \theta_{e}<\frac{5\pi }{3}\\ -1+\frac{6}{\pi }(\theta_{e}-\frac{5\pi }{3}) & \frac{5\pi }{3}\leq \theta_{e}<2\pi \end{array}\right.$$

**Figure 7:**
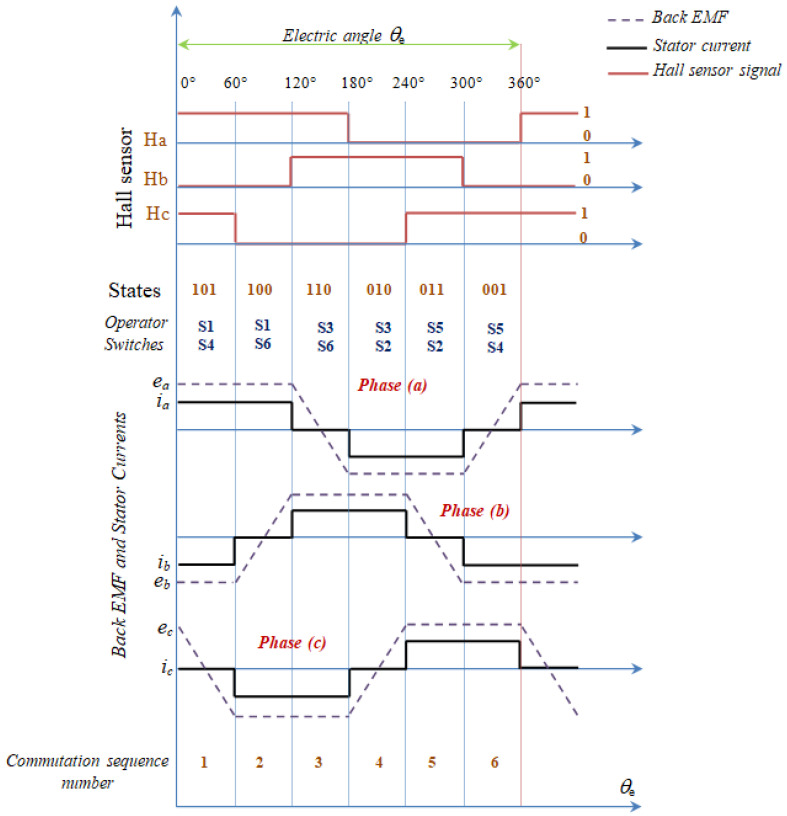
State of Hall sensor signals, Back-EMF and currents [47].

For illustration, Figure 7 shows Back-EMF, Hall Effect sensor signal and the current in the three phases. In the trapezoidal motor Back-EMF induced in the stator has a trapezoidal shape and its phases must be supplied with quasi square currents for ripple free torque operation [44,46]. 

Finally, Equations (15)–(18) can be converted to a state space form. The resulting complete model is given as:

(24) $$\left[\begin{array}{c} \frac{di_{a}}{dt}\\ \frac{di_{b}}{dt}\\ \begin{array}{c} \frac{d\omega_{m}}{dt}\\ \frac{d\theta_{m}}{dt} \end{array} \end{array}\right]=\left[\begin{array}{cccc} -\frac{R}{L} & 0 & 0 & 0\\ 0 & -\frac{R}{L} & 0 & 0\\ 0 & 0 & -\frac{k_{f}}{J} & 0\\ 0 & 0 & 1 & 0 \end{array}\right]\left[\begin{array}{c} i_{a}\\ i_{b}\\ \begin{array}{c} \omega_{m}\\ \theta_{m} \end{array} \end{array}\right]+\left[\begin{array}{ccc} \frac{2}{3L} & \frac{1}{3L} & 0\\ -\frac{1}{3L} & \frac{1}{3L} & 0\\ 0 & 0 & \frac{1}{J}\\ 0 & 0 & 0 \end{array}\right]\left[\begin{array}{c} v_{ab}-e_{ab}\\ v_{bc}-e_{bc}\\ T_{e}-T_{L} \end{array}\right]$$

(25) $$i_{c}=-(i_{a}+i_{b})$$

where: $e_{ab}=e_{a}-e_{b}$ and $e_{bc}=e_{b}-e_{c}$

### 4.3. Hall Effect Sensor and Transistor Switching Sequence 

According to the angular position of the rotor evolution between 0° and 360°, the position produced by Hall Effect sensors is given which is described in Table 5 below.

Each Hall Effect sensor operates during the passage of the poles based on the rising and falling edges. Thus, the rising front for the north pole and falling for the south pole. Accordingly, the sensor indicates 1 or 0, respectively. Following this switching logic of Hall Effect sensors, the switching sequence of the inverter is expressed in Table 5, where the switching sequence for shaft rotation is clockwise [45,47].

According to the circuit in Figure 6, the three-phase voltages are calculated with the following formulas [45]:

(26) $$v_{a}=\frac{v_{d}}{2}\left(S1-S2\right)$$

(27) $$v_{b}=\frac{v_{d}}{2}\left(S3-S4\right)$$

(28) $$v_{c}=\frac{v_{d}}{2}\left(S5-S6\right)$$

where *v*_d_ is the DC supply voltage.

### 4.4. Speed Control of Brushless DC Motor

**Figure 8:**
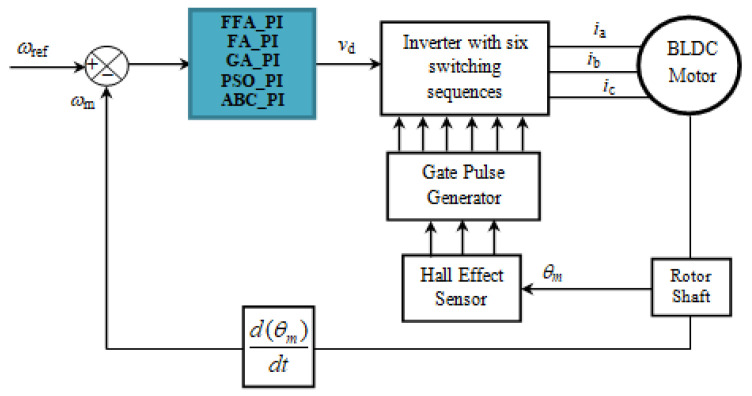
Speed control principle of BLDC motor.

The principle diagram for speed control of the three-phase BLDC motor is shown in Figure 8. At the regulator input, the reference speed is compared to the actual speed of the motor to generate a control voltage at its output.

The signals of the switching sequences are obtained from the position of the motor shaft. The motor stator is excited by the three-phase currents [45].

### 4.5. PI Controller 

PI controller is a derivative of PID controller. It has been extensively used in industrial applications due to its simplicity, robustness, reliability and easy tuning gains in simple control [21].

The equation of the PI controller is specified by:

(29) $$y\left(t\right)=k_{p}\varepsilon \left(t\right)+k_{i}\overset{t}{\underset{0}{\int }}\varepsilon \left(\tau \right)d\tau$$

The Laplace transfer function is:

(30) $$C\left(S\right)=k_{p}+\frac{k_{i}}{s}$$

where:

*k_p_*: proportional gain,

*k_i_*: integral gain,

*s*: Laplace operator.

### 4.6. Simulation Results and Discussion 

To ensure efficient performance of the system to be monitored, the performance criteria defined by Equations (31)–(34) are used. The objective functions are chosen for minimizing the time response characteristics due to the dependency of error on time [27]:

(31) $$J_{1}=IAE=\int_{0}^{T}\varepsilon (t)dt=\int_{0}^{T}(\omega_{ref}-\omega_{m})dt$$

(32) $$J_{2}=ISE=\int_{0}^{T}\varepsilon^{2}(t)dtdt=\int_{0}^{T}{\left(\omega_{ref}-\omega_{m}\right)}^{2}dt$$

(33) $$J_{3}=ITAE=\int_{0}^{T}t.\varepsilon (t)dt=\int_{0}^{T}t.(\omega_{ref}-\omega_{m})dt$$

(34) $$J_{4}=ITSE=\int_{0}^{T}t.\varepsilon^{2}(t)dt=\int_{0}^{T}t.{\left(\omega_{ref}-\omega_{m}\right)}^{2}dt$$

The problem can be represented as:

Minimize J subjected to:

*k_pmin_* ≤ *kp* ≤ *k_pmax_*

**Figure 9:**
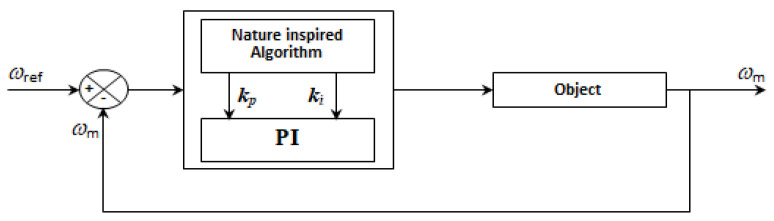
Scheme of PI controller parameters’ optimization based on a nature-inspired algorithm.

*k_imin_* ≤ *ki* ≤ *k_imax_*

where *ω*_ref_ is the reference speed and *ω*_m_ is the actual one. Figure 9 shows PI controller block of the control. In this problem, the values of overshoot, rise time and stabilization time are controlled indirectly. These parameters are directly linked to the objective function so they are optimized implicitly [27].

The model of BLDC motor drive is simulated in MATLAB. The parameters of the BLDC motor are reported in Table 6. 

To control the BLDC motor, a conventional PI controller is used. However, it is not easy to adjust its parameters in order to have an efficient control. Therefore, the FFA_PI controller is used and it is compared to other algorithms to evaluate its competitiveness. The simulation is performed by considering the well-known algorithms GA, PSO, ABC and the standard FA. The simulation is run with 100 iterations and a population size of 10. 

**Figure 10:**
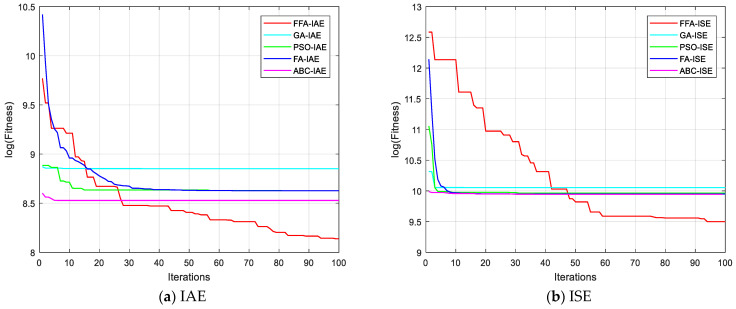

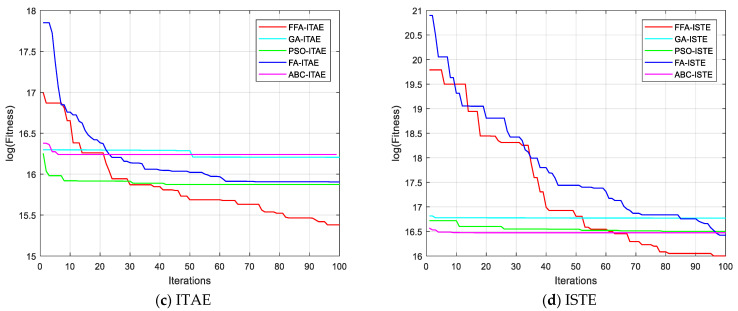
Convergence curves for different algorithms with several criteria.

**Figure 11:**
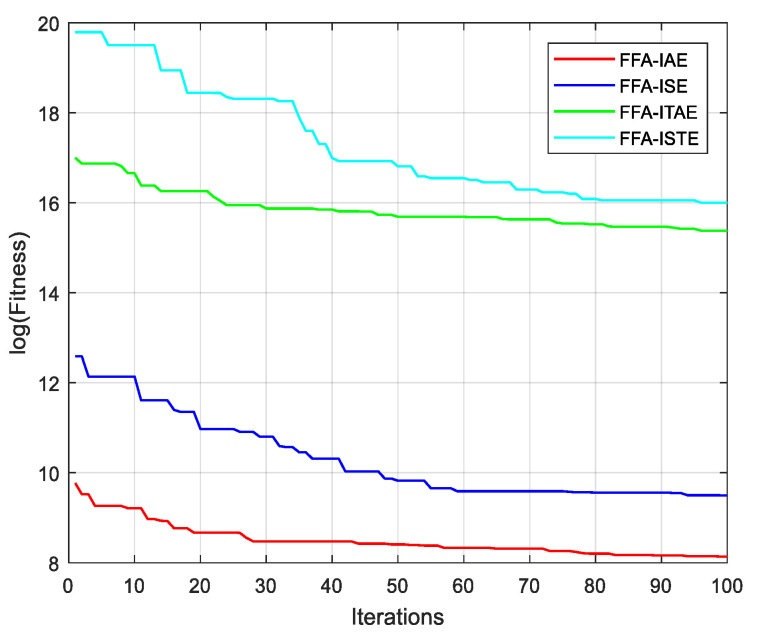
Convergence curves for different criteria with FFA algorithms.

Figure 10 shows the evolution of the different performance criteria with the different algorithms. The results of FFA, with the different criteria, are all the better than those presented by the other algorithms. Figure 11, also, presents the cost functions IAE, ISE, ITAE and ISTE obtained by FFA algorithm. 

The values of the PI controller, obtained by different simulations, are shown in Table 7. The values are obtained by the five algorithms used, with different criteria.

In the chosen cost functions, the values of the overshoot, the rise time and the settling time can be controlled indirectly. Based on their optimization, the cost functions force the values of the other parameters to be optimum [27]. Table 8 shows the values of the different correctors used in this simulation. The values of the rise time, settling time, peak time, peak and overshoot are reported in Table 8. Accordingly, the results concerning the time are better for the FFA algorithm as well as for the peaks and the overshoots which are alternated with the other algorithms. 

Moreover, the execution simulation time comparison is given between the different correctors and shown in Table 9. It can be reported that the calculation time using the FFA_PI is faster than those obtained with the FA_PI, GA_PI, PSO_PI and ABC_PI when using 50 or 100 iterations.

**Figure 12:**
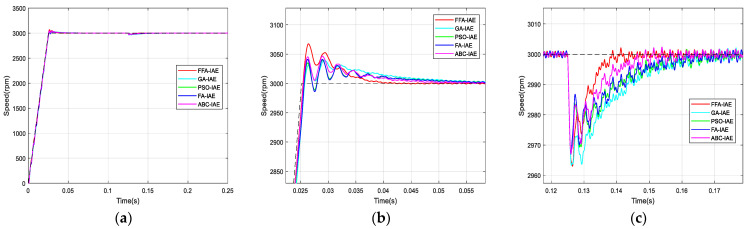
Comparison of speed responses with different algorithms using IAE criterion: (**a**) original; (**b**) and (**c**) zoomed version.

**Figure 13:**
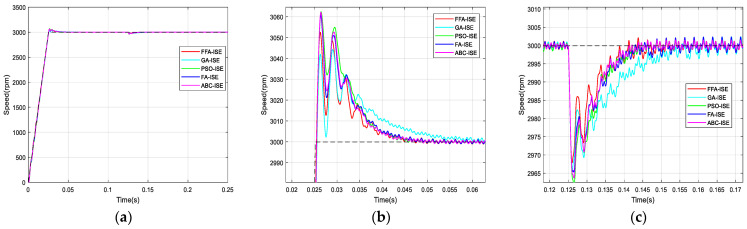
Comparison of speed responses with different algorithms using ISE criterion: (**a**) original; (**b**) and (**c**) zoomed version.

**Figure 14:**
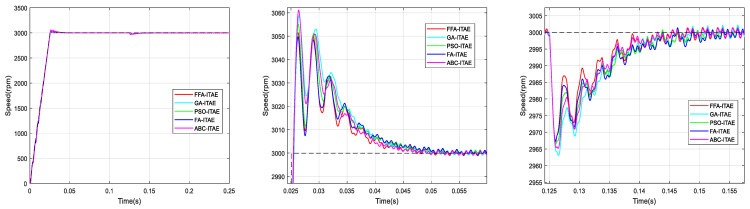
Comparison of speed responses with different algorithms using ITAE criterion: (**a**) original; (**b**) and (**c**) zoomed version.

**Figure 15:**
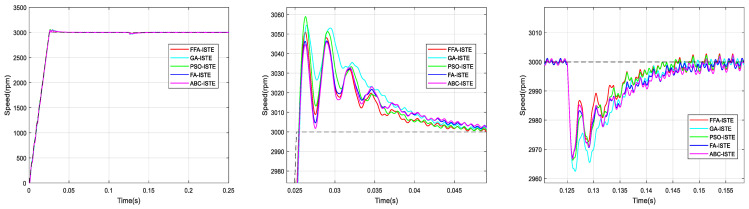
Comparison of speed responses with different algorithms using ISTE criterion: (**a**) original; (**b**) and (**c**) zoomed version.

According to the used criterions, Figure 12, Figure 13, Figure 14 and Figure 15 represent the BLDC motor speeds obtained with the different corrector optimized. Consequently, the figures are given for comparison and they justify the values in Table 8.

The graphs are zoomed in the area of the overshoot and the rejection of the disturbance for better visualization of signals. 

From the previous numerical results and the figures’ responses, it can be concluded that the optimized PI controller-based FFA showed a better capacity to compete with its FA counterpart, and its rivals GA, PSO and ABC. Thus, it provided the fastest rise and response times in addition to the minimum peak time.

**Figure 16:**
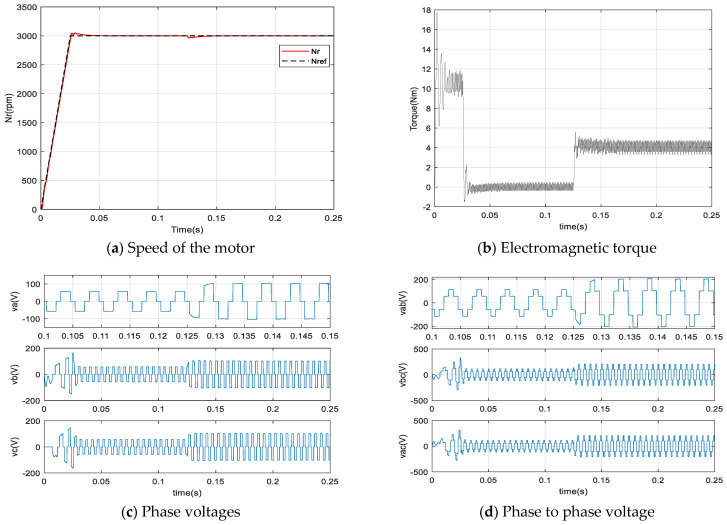

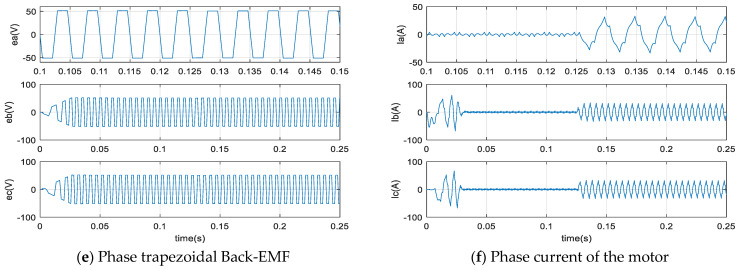
Results of simulation by using FFA_PI controller.

Figure 16 show the simulation results of the various variables of the BLDC motor using the FFA_PI using (ki = 2468, kp = 18.19). Accordingly, Figure 16a presents the speed of the BLDC motor where the reference speed ωref is chosen as a ramp in order to dampen the current at start-up and to avoid peaks as well as for the electromagnetic torque on the Figure 16b. At 0.125 s, a torque load TL = 4 Nm is applied and a good rejection by the control is observed. The effect of the load is very apparent on the figure of the speed, the torque, the voltages and the current.

**Figure 17:**
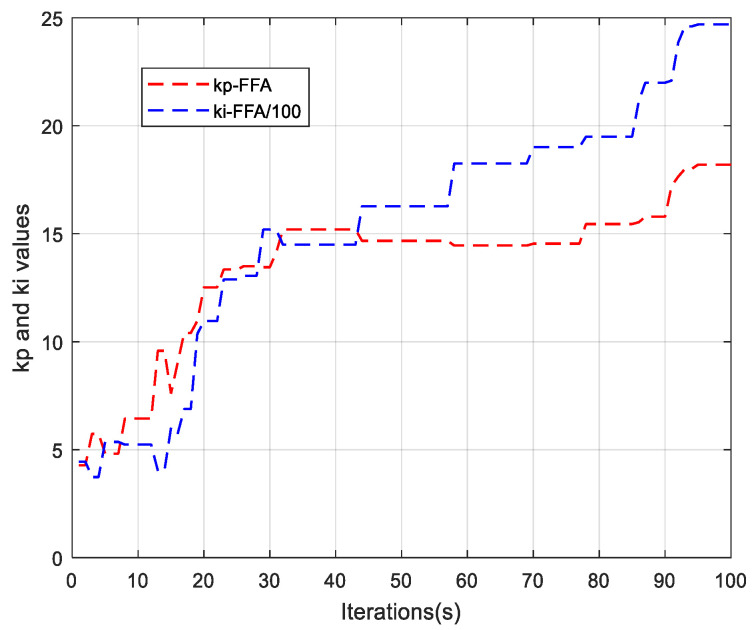
Evolution of parameters of FFA_PI until convergence.

On each figure presented, there are three phases, where the first phase is zoomed-in to clearly visualize the behavior of the signals. Thus, Figure 16c,d show the phase voltages and the phase to phase voltage simultaneously. The trapezoidal Back-EMF shape is well illustrated on the Figure 16e. Finally, the shape of the currents of the three phases of the stator is given by the Figure 16f. As can be seen, there is a distortion in the torque signals which is due to the trapezoidal shape of the Back-EMF and the nature of the currents containing harmonics. Finally, Figure 17 gives the evolution, until the convergence, of the parameters of the FFA_PI and FA_PI on the control technique.

## 5. Conclusions

A fast FA algorithm so-called FFA is presented and compared with the standard FA through searching the global optimum by using different standard benchmark functions in a first application. The simulation results were compared, taking in consideration the precision and the speed of convergence criteria for the two algorithms. The reached results prove that those obtained by FFA are better than those of FA. A second application concerning the optimization of the gains of a PI controlling a BLDC motor is carried out through the ITSE performance criterion. The results obtained show the robustness of the two algorithms with superiority for FFA. The acceleration of the proposed algorithm is due to the search space reduction by a random election of a significantly small set of moving fireflies while the whole search space stills covered. It should be noted that the acceleration, in the optimization function, is in the average 12:1, with respect to FA. Additionally, for the complex problem (BLDC motor control), the acceleration is clearly remarked for the modified algorithm FFA than FA, GA, PSO and ABC algorithms. Globally, the suggested FFA algorithm can be considered as most state of the art metaheuristic algorithms such as FA, GA, PSO and ABC, and presents superior fastness against all reported optimizers. 

Furthermore, a modification on the α parameter is given and this guarantees the robustness and precision through the enhancement of search directions toward the global optimal solution.

## Figures and Tables

**Table 1:** Benchmark functions.

Function	Name	Expression	Range	f(x*)
F1	Schaffer N.1	$F1\left(x,y\right)=\frac{\sin^{2}{\left(x^{2}+y^{2}\right)}^{2}-0.5}{{\left(1+0.001\left(x^{2}+y^{2}\right)\right)}^{2}}$	[−100,100]	0 for x* = (0,0)
F2	Matyas	$F2\left(x,y\right)=0.26\left(x^{2}+y^{2}\right)-0.48xy$	[−10,10]	0 for x* = (0,0)
F3	BohachevskyN1	$F3\left(x,y\right)=x^{2}+2y^{2}-0.3\cos(3\pi x)-0.4\cos(4\pi y)+0.7$	[−100,100]	0 for x* = (0,0)
F4	Xin-SheYang N.2	$F4\left(x\right)=\sum_{i=1}^{D}\left|x_{i}\right|\exp\left(\sum_{i}^{D}\sin\left(x_{i}^{2}\right)\right)$	[−2π,2π]	0 for x* = (0,……,0)
F5	Zakharov	$F5\left(x\right)=\sum_{i=1}^{D}x_{i}^{2}+{\left(\sum_{i=1}^{D}0.5ix_{i}\right)}^{2}+{\left(\sum_{i=1}^{D}0.5ix_{i}\right)}^{4}$	[−5,10]	0 for x* = (0,……,0)
F6	Ackley	$F6\left(x\right)=-20\exp(-0.2\sqrt{\frac{1}{D}\sum_{i=1}^{D}x_{i}^{2}})-$ $\;\;\;\;\;\;\exp(\frac{1}{D}\sum_{i=1}^{D}\cos(2\pi x_{i})+20+\exp(1)$	[−32,32]	0 for x* = (0,……,0)
F7	Powell	$F7\left(x\right)=\sum_{i=1}^{D}{\left|x_{i}\right|}^{i+1}$	[−1,1]	0 for x* = (0,……,0)
F8	Rastrigin	$F8\left(x\right)=10D+\sum_{i=1}^{D}\left[x_{i}^{2}-10\cos(2\pi x_{i})\right]$	[−5.12,5.12]	0 for x* = (0,……,0)
F9	Schewel223	$F9\left(x\right)=\sum_{i=1}^{D}x_{i}^{10}$	[−10,10]	0 for x* = (0,0)
F10	Alpinen1	$F10\left(x\right)=\sum_{i=1}^{D}\left|x_{i}\sin(x_{i})+0.1x_{i}\right|$	[0,10]	0 for x* = (0,0)
F11	Grienwak	$F11\left(x\right)=1+\sum_{i=1}^{D}\frac{x_{i}^{2}}{4000}-\prod_{i=1}^{D}\cos\left(\frac{x_{i}}{\sqrt{i}}\right)$	[−600,600]	0 for x* = (0,……,0)
F12	Brown	$F12\left(x\right)=\sum_{i=1}^{D-1}{\left(x_{i}^{2}\right)}^{\left(x_{i+1}^{2}+1\right)}+{\left(x_{i+1}^{2}\right)}^{\left(x_{i}^{2}+1\right)}$	[−1,4]	0 for x* = (0,……,0)
F13	Sphere	$F13\left(x\right)=\sum_{i=1}^{D}x_{i}^{2}$	[−5.12,5.12]	0 for x* = (0,……,0)
F14	Salomon	$F14\left(x\right)=1-\cos\left(2\pi \sqrt{\sum_{i=1}^{D}x_{i}^{2}}\right)+0.1\sqrt{\sum_{i=1}^{D}x_{i}^{2}}$	[−100,100]	0 for x* = (0,……,0)
F15	Three Hump Camel	$F15\left(x\right)=2x_{1}^{2}-1.05x_{1}^{4}+\frac{x_{1}^{6}}{6}+x_{1}x_{2}+x_{2}^{2}$	[−5,5]	0 for x* = (0,……,0)

**Table 2:** Parameter settings of FA and FFA.

Symbol	Quantity	Value
N	Population size	30
*Iter*	Number of iterations	1000
*α*	Randomization parameter	[0,1]
*β*_0_	Attractiveness	1
*γ*	Absorption coefficient	[0,1]

**Table 3:** Comparative simulation results of FA and FFA for the 12 benchmark test functions.

Function	Algorithm	Dim. D	Theoretical Optimal Value	Minimum Value	Computational Time (s)	Average Speed up Ratio of 10 Runs	Std	Mean
F1	FA	2	0	1.6542 × 10^−13^	86.483887	12.0295:1	2.9426 × 10^−12^	3.4053 × 10^−12^
	FFA			2.2204 × 10^−16^	7.189327		1.1466 × 10^−16^	3.5527 × 10^−16^
F2	FA	2	0	5.8618 × 10^−15^	110.617901	11.7869:1	2.2829 × 10^−15^	4.2810 × 10^−15^
	FFA			1.1794 × 10^−22^	9.384792		5.2167 × 10^−22^	3.4229 × 10^−22^
F3	FA	2	0	8.0766 × 10^−10^	114.399397	11.9121:1	2.2452 × 10^−9^	2.2950 × 10^−9^
	FFA			1.1102 × 10^−16^	9.603642		1.0320 × 10^−15^	1.4211 × 10^−15^
F4	FA	10	0	5.6623 × 10^−4^	165.318332	13.0661:1	1.3542 × 10^−8^	5.6625 × 10^−8^
	FFA			3.4134 × 10^−11^	12.652510		2.4298 × 10^−11^	3.6237 × 10^−11^
F5	FA	10	0	1.9635 × 10^−7^	102.111224	12.0521:1	1.1191 × 10^−7^	3.1165 × 10^−7^
	FFA			5.0686 × 10^−22^	8.472478		9.9126 × 10^−38^	5.0686 × 10^−22^
F6	FA	10	0	0.0252	174.645121	12.6941:1	0.0061	0.0357
	FFA			7.5286 × 10^−11^	13.758015		5.5179 × 10^−11^	8.6060 × 10^−11^
F7	FA	20	0	5.4225 × 10^−8^	254.090816	11.9330:1	4.4293 × 10^−8^	6.0622 × 10^−8^
	FFA			6.0701 × 10^−27^	21.293091		1.6065 × 10^−25^	2.1326 × 10^−25^
F8	FA	20	0	1.7397 × 10^−9^	86.135286	11.9768:1	2.2366 × 10^−9^	2.5533 × 10^−9^
	FFA			7.1054 × 10^−15^	7.191834		1.1235 × 10^−14^	1.0658 × 10^−14^
F9	FA	20	0	5.4774 × 10^−26^	199.923385	12.2129:1	6.8916 × 10^−26^	7.4821 × 10^−26^
	FFA			2.9153 × 10^−84^	16.369803		4.8193 × 10^−100^	2.9153 × 10^−84^
F10	FA	30	0	1.7988 × 10^−4^	139.507606	12.2676:1	1.5871 × 10^−5^	2.1766 × 10^−4^
	FFA			5.6687 × 10^−9^	11.372018		3.6465 × 10^−10^	6.0921 × 10^−9^
F11	FA	30	0	8.0295 × 10^−6^	130.429223	17.9004:1	2.0834 × 10^−7^	1.2770 × 10^−6^
	FFA			3.3304 × 10^−16^	7.286405		2.0572 × 10^−16^	6.5503 × 10^−16^
F12	FA	30	0	2.0832 × 10^−4^	312.021582	16.2503:1	2.1256 × 10^−5^	1.7312 × 10^−4^
	FFA			3.5141 × 10^−16^	19.200945		2.1964 × 10^−17^	3.5897 × 10^−16^

**Table 4:** Stability of FFA in higher dimensions.

Function	Algorithm	Dim. D	Theoretical Optimal Value	Minimum Value	Std	Mean	Computational Time of 10 Runs (Seconds)	Iterations
F13	FFA	50	0	3.7950 × 10^−16^	2.9222 × 10^−17^	3.9330 × 10^−16^	11.743543	1000
		100		8.9766 × 10^−16^	5.3287 × 10^−17^	9.2937 × 10^−16^	12.630883	
		150		1.5083 × 10^−15^	4.4389 × 10^−17^	1.5223 × 10^−15^	13.399011	
		200		2.1964 × 10^−15^	8.7426 × 10^−20^	2.1966 × 10^−15^	14.696007	
F14	FFA	50	0	1.9615 × 10^−9^	5.8730 × 10^−11^	2.0092 × 10^−9^	12.619130	1000
		100		3.0618 × 10^−9^	6.2137 × 10^−11^	3.1208 × 10^−9^	13.483702	
		150		3.9315 × 10^−9^	1.7846 × 10^−11^	3.9107 × 10^−9^	14.451983	
		200		4.6921 × 10^−9^	1.1344 × 10^−11^	4.6959 × 10^−9^	15.718014	
F15	FFA	50	0	1.1834 × 10^−21^	7.7571 × 10^−22^	2.0847 × 10^−21^	8.676127	1000
		100		1.3134 × 10^−21^	9.5957 × 10^−22^	1.7686 × 10^−21^	9.238064	
		150		1.6926 × 10^−21^	4.3289 × 10^−21^	3.9281 × 10^−21^	10.033184	
		200		2.8146 × 10^−21^	3.7922 × 10^−21^	5.1335 × 10^−21^	11.103394	

**Table 5:** Switching sequence by using Hall Effect sensor signals.

Electrical Angle (°)	Sequence Number	Hall Sensors			Phase Current			Switch Closed	
		Ha	Hb	Hc	ia	ib	ic		
0–60	1	1	0	1	+	−	off	S1	S4
60–120	2	1	0	0	+	off	−	S1	S6
120–180	3	1	1	0	off	+	−	S3	S6
180–240	4	0	1	0	−	+	off	S3	S2
240–300	5	0	1	1	−	off	+	S5	S2
300–360	6	0	0	1	off	−	+	S5	S4

**Table 6:** Parameters of BLDC motor.

Parameters	Values
Number of pole	4
Nominal voltage *vd*	114 V
Stator resistance *R*	1.2 Ω
Stator inductance *L*	1.2 mH
Torque coefficient *k_t_*	0.3262 Nm/A
Back-EMF coefficient *k_e_*	0.3262 Vs/rad
Rotor inertia J	0.00085 kgm^2^
Rated speed Nr	3000 rpm
Friction coefficient *k_f_*	0.0001 Nms/rad

**Table 7:** kp, ki parameters obtained by various objective functions and various algorithms.

Algorithm	Parameters/Criterion	*IAE*	*ISE*	*ITAE*	*ITSE*
FFA	kp_FFA	18.19	24.5	24.56	24.06
	ki_FFA	4468.8	4435.2	4132.2	4002.32
FA	kp_FA	26.54	20.21	24.08	24.08
	ki_FA	2207.2	3615.8	3451.2	2996.2
GA	kp_GA	19.45	23.14	19.11	24.76
	ki_GA	1685.1	2474.5	3220.2	2896.1
PSO	kp_PSO	25.8	17.68	22.06	21.72
	ki_PSO	2081.5	3440.8	3451.2	3601.66
ABC	kp_ABC	24.51	19.53	20.16	24.17
	ki_ABC	3140.9	3796.6	3650.8	2901.12

**Table 8:** Performance of the different controllers.

Controller	Criterion	Rise Time(s)	Settling Time(s)	Peak	Peak Time(s)	% Overshoot
FFA_PI	IAE	**0.0217**	**0.413**	3067.3	**0.0264**	2.2497
FA_PI		0.0219	0.0722	3041.1	0.0289	1.3708
GA_PI		0.0219	0.0731	**3038.8**	0.0293	**1.2922**
PSO_PI		0.0219	0.0730	3039.9	0.0289	1.3299
ABC_PI		0.0219	0.0579	3046.5	0.0289	1.5486
FFA_PI	ISE	**0.0217**	**0.0478**	**3058.2**	**0.0263**	1.7516
FA_PI		0.0218	0.0488	3060.7	0.0264	2.0226
GA_PI		0.0219	0.0646	3044.3	0.0290	**1.4769**
PSO_PI		**0.0217**	0.0488	3062.4	0.0265	2.0804
ABC_PI		**0.0217**	0.0488	3062.1	0.0264	2.0713
FFA_PI	ITAE	**0.0218**	**0.0488**	3051.5	**0.0263**	1.7163
FA_PI		0.0219	0.0546	**3049.7**	**0.0263**	**1.6554**
GA_PI		**0.0218**	**0.0488**	3058.9	0.0264	1.9620
PSO_PI		0.0219	0.0612	3055.2	**0.0263**	1.8398
ABC_PI		**0.0218**	**0.0488**	3061.2	0.0264	2.0393
FFA_PI	ISTE	**0.0218**	**0.0513**	3051.0	**0.0263**	1.7013
FA_PI		0.0219	0.0613	3046.4	0.0290	1.5480
GA_PI		0.0219	0.0613	3046.4	0.0290	**1.5473**
PSO_PI		**0.0218**	**0.0513**	3058.9	**0.0263**	1.9625
ABC_PI		0.0219	0.0596	**3045.8**	0.0290	1.5269

**Table 9:** Simulation time of the five algorithms.

	Simulation Time (s)				
Iteration	FFA	FA	GA	PSO	ABC
50	108.53	284.15	119.55	242.23	135.95
100	216.57	570.06	239.77	486.82	268.20

## Nomenclature

Symbols used in this paper	
ABC	Artificial Bee Colony
PSO	Particle Swarm Optimization
CS	Cuckoo Search
BA	Bat Algorithm
GWO	Gray Wolf Optimizer
FA	Firefly Algorithm
FFA	Fast Firefly Algorithm
GA	Genetic Algorithms
PI	Proportional Integral
PID	Proportional Integral & Derivative
*kp*, *ki*	Proportional and Integral gains of the PI controller
IFA	Improved Firefly Algorithm
MFA	Modified Firefly Algorithm
*t_FA_*	Execution time of the original algorithm FA
*t_FFA_*	Execution time of the proposed algorithm FFA
*R*	Resistance of a stator phase, [Ω]
*L*	Inductance of a stator phase, [H]
*v_a_*, *v_c_*, *v_c_*	Stator phase voltages, [V]
*v_ab_*, *v_bc_*, *v_ca_*	Stator phase to phase voltages, [V]
*i_a_*, *i_b_*, *i_c_*	Stator phase currents, [A]
*e_a_*, *e_b_*, *e_c_*	Motor Back-EMFs, [V]
*T_e_*, *T_L_*	Electromagnetic and load torques, [Nm]
*J*	Rotor inertia, [kgm^2^]
*k_f_*	Friction constant, [Nms/rad]
*kt*	Torque coefficient, [Nm/A]
*ω_m_*	Rotor speed, [rad/s]
Nr	Rated speed, [rpm]
*θ_e_*	Electric angle of rotor, [rad]
*θ_m_*	Mechanic angle of rotor, [rad]
*F(θ_e_)*	Back-EMF reference function
*ε(t)*	Error input signal
*y(t)*	Output signal
Ha, Hb, Hc	Hall Effect Sensors for the three phases
*IAE*	Integral Absolute Error
*ISE*	Integral Square Error
*ITAE*	Integral Time Absolute Error
*ISTE*	Integral Square Time Error
